# Enhancing 2D Photonics and Optoelectronics with Artificial Microstructures

**DOI:** 10.1002/advs.202403176

**Published:** 2024-06-21

**Authors:** Haizeng Song, Shuai Chen, Xueqian Sun, Yichun Cui, Tanju Yildirim, Jian Kang, Shunshun Yang, Fan Yang, Yuerui Lu, Linglong Zhang

**Affiliations:** ^1^ Henan Key Laboratory of Rare Earth Functional Materials Zhoukou Normal University Zhoukou 466001 China; ^2^ College of Physics, Nanjing University of Aeronautics and Astronautics Key Laboratory of Aerospace Information Materials and Physics (NUAA), MIIT Nanjing 211106 China; ^3^ School of Engineering, College of Engineering and Computer Science the Australian National University Canberra ACT 2601 Australia; ^4^ National Key Laboratory of Science and Technology on Test Physics and Numerical Mathematics Beijing 100190 China; ^5^ Faculty of Science and Engineering Southern Cross University East Lismore NSW 2480 Australia; ^6^ Laboratory of Solid State Microstructures Nanjing University Nanjing 210093 China

**Keywords:** 2D artificial microstructures, heterostructures, integrations, metasurfaces, optoelectronics

## Abstract

By modulating subwavelength structures and integrating functional materials, 2D artificial microstructures (2D AMs), including heterostructures, superlattices, metasurfaces and microcavities, offer a powerful platform for significant manipulation of light fields and functions. These structures hold great promise in high‐performance and highly integrated optoelectronic devices. However, a comprehensive summary of 2D AMs remains elusive for photonics and optoelectronics. This review focuses on the latest breakthroughs in 2D AM devices, categorized into electronic devices, photonic devices, and optoelectronic devices. The control of electronic and optical properties through tuning twisted angles is discussed. Some typical strategies that enhance light‐matter interactions are introduced, covering the integration of 2D materials with external photonic structures and intrinsic polaritonic resonances. Additionally, the influences of external stimuli, such as vertical electric fields, enhanced optical fields and plasmonic confinements, on optoelectronic properties is analysed. The integrations of these devices are also thoroughly addressed. Challenges and future perspectives are summarized to stimulate research and development of 2D AMs for future photonics and optoelectronics.

## Graphical abstract

This article overviews the progress in studies on 2D artificial microstructure devices (2D AMs), encompassing electronic devices, photonic devices, optoelectronic devices and device integrations. This article also discusses typical strategies to enhance light‐matter interactions and analyzes the influences of external stimuli on optoelectronic properties. Furthermore, it summarizes the challenges and future perspectives to stimulate research and development of 2D AMs for future photonics and optoelectronics.

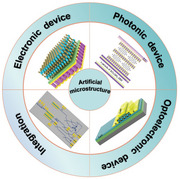

## 1 Introduction

Photonic and optoelectronic systems based on 2D artificial microstructures (2D AMs) efficiently control light by altering amplitude, phase, polarization states and doping levels. 2D AM modulation manipulates the electromagnetic wavefront controlling information transmission for sensing, imaging and communications in many fields.^[^ ^1^, ^2^, ^3^, ^4^, ^5^, ^6^, ^7^, ^8^, ^9^, ^10^ ^]^ In stark contrast with bulky and heavy counterparts, 2D AMs offer numerous advantages when integrated within photonic and optoelectronic devices. 2D AMs comprise subwavelength structures, such as metasurfaces, superlattices, heterostructures and microcavities.^[^ ^11^, ^12^, ^13^, ^14^, ^15^, ^16^, ^17^, ^18^, ^19^, ^20^ ^]^ Among them, metasurfaces consist of single or multi‐layer planar stacking structures, which are easily fabricated through lithograph and nanoimprinting processes. Moreover, metasurfaces reveal dependency between transmission and reflection with surface and interface, regulating surface impedance, where metasurfacesʼ permittivity, permeability and refractive index originate from the metamaterials.^[^ ^21^ ^]^ Thus, metasurfaces provide a solution to overcome metamaterialsʼ drawbacks, including high loss, strong dispersion, and complicated fabrication processes, while simultaneously achieving strong interactions between incident waves.

**Figure 1:**
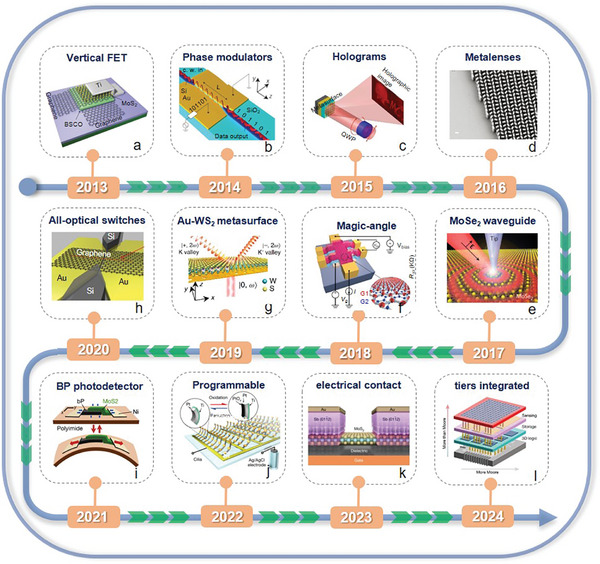
The timeline of typical 2D AM devices. a) Reproduced with permission.^[^ ^33^ ^]^ Copyright 2013, Springer Nature. b) Reproduced with permission.^[^ ^34^ ^]^ Copyright 2014, Springer Nature. c) Reproduced with permission.^[^ ^29^ ^]^ Copyright 2015, Springer Nature. d) Reproduced with permission.^[^ ^28^ ^]^ Copyright 2016, The American Association for the Advancement of Science. e) Reproduced with permission.^[^ ^35^ ^]^ Copyright 2017, Springer Nature. f) Reproduced with permission.^[^ ^30^ ^]^ Copyright 2018, Springer Nature. g) Reproduced with permission.^[^ ^22^ ^]^ Copyright 2019, Springer Nature. h) Reproduced with permission.^[^ ^36^ ^]^ Copyright 2020, Springer Nature. i) Reproduced with permission.^[^ ^24^ ^]^ Copyright 2021, Springer Nature. j) Reproduced with permission.^[^ ^25^ ^]^ Copyright 2022, Springer Nature. k) Reproduced with permission.^[^ ^40^ ^]^ Copyright 2023, Springer Nature. l) Reproduced with permission.^[^ ^2^ ^]^ Copyright 2024, Springer Nature.

In the last ten years, 2D AM‐based photonic and optoelectronic devices have made much progress, and **Figure** **1** summarizes some typical works in the timeline.^[^ ^22^, ^23^, ^24^, ^25^, ^26^, ^27^, ^28^, ^29^, ^30^, ^31^ ^]^ In 2011, Yu et al. designed a 2D optical resonator array with subwavelength separation and spatially variable phase.^[^ ^27^ ^]^ As light traverses two media interfaces, it can impose phase discontinuities in the traveling wave. Lee et al. implemented substantial gate‐induced persistent switching and linear modulation of terahertz waves using a 2D metamaterial incorporating an atomically thin graphene layer which is gate‐tunable.^[^ ^32^ ^]^ In 2013, a vertical field‐effect transistor (VFET) was fabricated with a high on/off ratio and current density of cm^−2^ (Figure 1a).^[^ ^33^ ^]^ Moreover, Melikyan et al. designed a high‐speed plasma phase modulator based on the Pockels effect. The device has an ultrasmall size and super high operation rate (Figure 1b).^[^ ^34^ ^]^ Zheng et al. brought together a grounded metal plane and a geometric metasurface to implement a geometric metasurface hologram (Figure 1c). It not only significantly improves the conversion efficiency between the two circular polarization states, but also achieves a high diffraction efficiency without complicated fabrications.^[^ ^29^ ^]^ Khorasaninejad et al. reported high‐aspect‐ratio titanium dioxide based metalenses, exhibiting a nanoscale‐level resolution and achieving 170× magnification for state‐of‐the‐art imaging (Figure 1d).^[^ ^28^ ^]^ In addition, Hu et al. examined nano optical imaging by using thin transition metal dichalcogenide (TMDC) planar waveguides, where the strong coupling between excitons and waveguide photons forms exciton‐polaritons (EPs). Their propagation length is sensitive to excitation photon energies over 12 µm (Figure 1e).^[^ ^35^ ^]^ The wavelength of the polaron is easily tuned from 600 to 300 nm through alterations in the thickness of the waveguide. Recently, heterostructures or superlattices have emerged as the critical component for contemporary photonics and optoelectronics, since they inherit the merits of constituent layers as well as trigger new physical properties. Cao et al. observed unconventional superconductivity induced via twist‐angle graphene superlattices. At the near zero Fermi energy, the flat band appears, indicating the correlated insulating states at the half filling (Figure 1f).^[^ ^30^ ^]^ Hu et al. demonstrated a synthetic metasurface that entangles the phase and spin of light simultaneously. This structure enhances and manipulates the nonlinear valley‐locking chiral emission of monolayer tungsten disulfide (WS_2_).^[^ ^22^ ^]^ Meanwhile, second‐harmonic valley photons and coherent pumping are endowed through a gold (Au) metasurface with spin‐dependent geometric phases. Photons are separated and follow in the way of the specific direction in free space. (Figure 1g). On the other hand, all‐optical switches have risen as a star for the capability of surpassing the rate limits of electronic switches. However, implementing ultrafast and energy‐efficient all‐optical switches is still lacking, which can be attributed to limited optical nonlinearity in existing materials. In 2020, Masaaki et al. applied graphene‐loaded deep subwavelength plasmonic waveguides to greatly enhance optical nonlinear absorption in graphene, which is beneficial to achieve an ultrafast all‐optical switch (Figure 1h).^[^ ^36^ ^]^ The energy required for switching is reduced by four orders of magnitude compared to previous studies,^[^ ^37^, ^38^, ^39^ ^]^ marking this device as the minimum all‐optical switch within a timeframe of a few picoseconds or shorter. Kim et al. fabricated light‐emitting diodes (LEDs) and photodetectors based on black phosphorus (BP), which can continuously and reversibly tune the operation wavelengths (Figure 1i).^[^ ^24^ ^]^ Recently, Wang et al. realized an active metasurface of electronically driven artificial cilia that can generate arbitrary flow patterns in fluids close to the surface (Figure 1j).^[^ ^25^ ^]^ Li et al. realized near‐quantum‐limit electrical contact between MoS_2_ and semi‐metallic Sb, the contact exhibited excellent electrical performance, stability and variability (Figure 1k).^[^ ^40^ ^]^ Jayachandran et al. demonstrated 3D integration of 2D field‐effect transistors in each tier and achieved sensing and storage capabilities (Figure 1l).^[^ ^2^ ^]^ To further develop these growing fields, it is scientifically important and pressing to summarize recent breakthroughs of 2D AMs and their corresponding photonic and optoelectronic device applications.

The latest progress of 2D AM‐based devices is first summarized and categorized into electronic devices, photonic devices, and optoelectronic devices. Second, 2D AM device integrations are introduced in detail. Finally, the future challenges and prospects are carefully analyzed for developing 2D AMs and related devices. This review aims to encourage more researchers to join this field and contribute to the future potential of engineering light‐matter interactions at the nanometer scale.

## 2 Electronic Devices

In the past 60 years, various strategies have been used to improve electronic device performance, for example, decreasing dimensions, choosing new materials and geometries. However, unintended problems occur when scaling down device size, such as excessive leakage currents, short channel effects and complicated doping profiles.^[^ ^41^, ^42^ ^]^ Thus, 2D AMs have been proposed to overcome scaling issues, while reducing consumption and cost.^[^ ^30^, ^41^, ^43^, ^44^, ^45^, ^46^, ^47^, ^48^, ^49^, ^50^, ^51^, ^52^, ^53^, ^54^, ^55^, ^56^, ^57^ ^]^ Research into 2D AM‐based electronics has covered transistors and their related functional devices,^[^ ^52^, ^58^, ^59^, ^60^ ^]^ including p‐n heterostructures,^[^ ^61^ ^]^ image sensors,^[^ ^62^ ^]^ displays,^[^ ^63^ ^]^ artificial skin,^[^ ^64^ ^]^ circuits and so on.^[^ ^65^ ^]^

**Figure 2:**
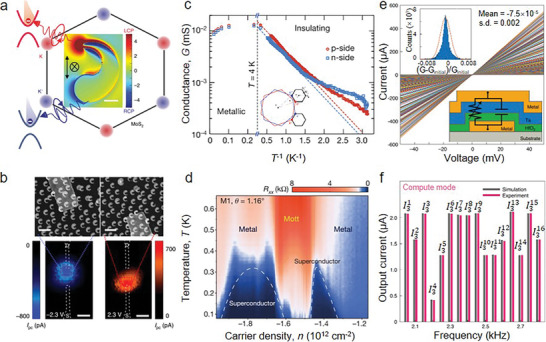
2D AM electronic applications. a) The calculation of local enhancement of the proposed valleytronic transistor based on Chiral nanocrescent plasmonic antennae and MoS_2_. b) Photocurrent mappings of the designed device under different bias polarities −2.3 and 2.3 V corresponding to left and right panels, respectively (Reproduced with permission.^[^ ^58^ ^]^ Copyright 2020, Springer Nature). c) Minimum conductance values in magic angle TBG device for p and n‐side half‐filling states corresponding to the red and blue curves. The inset figure shows the mini‐Brillouin zone (Reproduced with permission.^[^ ^43^ ^]^ Copyright 2018, Springer Nature). d) The density and temperature function for TBG devices’ four‐probe resistance (θ_*magic*_ = 1.1°) (Reproduced with permission.^[^ ^30^ ^]^ Copyright 2018, Springer Nature). e) The *I*–*V* curves of the Ta/HfO_2_ memristor crossbar array system at various conductance states, the inset top figure is the error distribution of equipment conductance and the bottom is the schematic of device structure. f) A comparison of experimental (magenta histogram) and simulated (grey histogram) current levels in the parallel computing mode at 16 frequencies (Reproduced with permission.^[^ ^77^ ^]^ Copyright 2021, Springer Nature).

2D semiconductors are a promising candidate for next‐generation information devices, due to an intrinsic valley degree of freedom.^[^ ^58^, ^66^ ^]^ Excitonic injection or the non‐local response of transverse current schemes under cryogenic temperature demonstrated the valleytronic properties as previously reported.^[^ ^67^, ^68^ ^]^ But low temperature limits applications utilizing the valley Hall effect. Li et al. reported a molybdenum disulfide (MoS_2_) valleytronic transistor which can realize the generation, propagation, detection, and manipulation of valley information at room temperature (**Figure** **2**a,b).^[^ ^58^ ^]^ This valley transistor contained a Hall‐bar structure, which is used for electrical read‐out of valley information. Moreover, it comprises asymmetric nanocrescent plasmonic antennae, allowing for artificial valley‐selective charge injections in MoS_2_. This phenomenon was observed under a 1550 nm excitation using a linearly polarized monochromatic light, due to its circular dichroism properties (Figure 2a). By using valley Hall configurations, the long‐lived valley polarized free carriers with a propagation length over 18 µm via drift were observed. Figure 2b depicts the photocurrent mappings under different bias polarities, where the current polarities and band bending substantiate the photo‐voltaic like carrier injection for photocurrents. The injection of free electrons maintains the disrupted time‐reversal symmetry in the plasmonic absorption. Different electron populations in the K valleys as well as valley polarization in MoS_2_ were investigated. In addition, the two asymmetric terms of the proposed plasmonic antennae, which are oriented toward the electrodes, are aligned in the experiments. However, a contrasting valley polarization was observed between the electrodes pumping. Valley polarization is confirmed by using circular‐polarization‐resolved photoluminescence (PL). PL polarization to uneven quantum yields in distinct valleys is attributed to the 1550 nm laser illumination‐induced valley‐selective doping. Moreover, Jiang et al. observed the robust ambient temperature valley Hall effect in MoS_2_/WSe_2_ heterostructures.^[^ ^69^ ^]^ The valley Hall effect's polarity and magnitude can be adjusted through gating, owing to the inverse valley Hall effect of holes and electrons in diverse layers. Zhou et al. fabricated a hetero‐dimensional superlattice structure (2D vanadium disulfide (VS_2_) and a 1D vanadium sulfide (VS) chain array) through a one‐step chemical vapor deposition method,^[^ ^70^ ^]^ which exhibit in‐plane anomalous Hall effect at ambient temperatures. Arrighi et al. showed the non‐identical moiré twins in bilayer graphene/BN (boron nitride) heterostructures, indicating distinct electronic properties and valley Hall effects at different alignments (0° and 60°), which are attributed to atomic structure relaxation.^[^ ^71^ ^]^ These differences demand further theoretical and experimental investigations. Therefore, the coming researchers can exploit the unequal distribution of electrons in different energy valleys to develop a new information encoding method, making valleys a new information carrier. These results facilitate the realization of new valleytronic devices with high computing speed, low power consumption and non‐volatility.

2D van der Waals (vdW) heterostructures that assemble through weak vdW forces not only inherit the merits of constituent layers but also create novel optoelectronic properties. These highly tunable properties supply a new avenue to enhance device performance. In particular, the twist angle between the adjacent layers has become an important route to tune their electronic properties. For instance, moiré patterns are produced by lattice misorientations at small twist angles, leading to the long‐range tuning of stacking orders. A flat band at near Fermi level was observed, which is attributed to the strong interlayer couplings at the magic angle (Figure 2c).^[^ ^43^ ^]^ Meanwhile, insulating states at half‐filling occur in these flat states, which is unexpected in uncorrelated electrons. They are ascribed to Mott‐like insulate states since the electrons are localized into moiré pattern induced superlattices. In addition, unconventional superconductivity was noticed in graphene superlattices at the twist angle of ≈1.1°, which was not due to weak electron‐phonon interactions (Figure 2d).^[^ ^30^ ^]^ On two sides of half‐filling correlated insulating states, the twisted bilayer graphene device exhibited two clear superconducting domes, which were similar to cuprate superconductivity at high temperatures. An ultralow resistance appears inside the domes and its values exceed twice as low as a standard device. While decreasing temperatures and fixing the carrier density in the middle‐half‐filling states, the correlated insulating phase occurs at ≈1 to 4 K. Superconductivity appears at the lowest temperatures (Figure 2d). Lee et al. devised a solid‐state ionic doping method for 2D semiconductors by utilizing the superionic phase transition of AgI.^[^ ^72^ ^]^ The devices could reversibly program into transistors or diodes that show device polarities or switchable carrier types. On the other hand, the reconfigurable inverters, NORs and NAND gates were constructed based on the above devices, and environmental cues can erase them. Zhao et al. demonstrated a universal way to fabricate higher‐order vdW superlattices through using different 2D vdW materials. These superlattices exhibited the designable band offset and the tunability of the carrier confinements or carrier separations.^[^ ^73^ ^]^ Liu et al. presented a new correlated insulator that evolved from spin‐polarized states to valley‐polarized states in twisted double bilayer graphene (TDBG) driven by the displacement field.^[^ ^74^ ^]^ New phases of matter are discovered, including valley‐polarized Chern insulators and valley‐polarized Fermi surfaces. Furthermore, they investigated the impact of these polarizations on the emergence of new electronic phases in TDBG. Kapfer et al. employed atomic force microscopy to bend 2D material ribbons, achieving continuous variation of twist angles and reduced moiré disorder. These results enable precise control over moiré superlattices, beneficial for the design of advanced quantum materials and devices.^[^ ^75^ ^]^ Craig et al. utilized an interferometric 4D scanning transmission electron microscopy approach to directly probe the local atomic stacking and symmetry in twisted graphene trilayers, revealing significant reconstruction and its implications for correlated electron behavior.^[^ ^76^ ^]^ The higher‐order and higher‐quality vdW superlattices with highly controllable twist angles lead to various exotic physical phenomena and new functionalities, offering new avenues for fundamental physics studies and technological applications.

In addition, a scalable massively parallel computing approach was proposed using a nanoscale Ta/HfO_2_ crossbar array, which realized both frequency multiplexing and continuous‐time data representation.^[^ ^77^ ^]^ Figure 2e illustrates the current‐voltage characteristics, linear *I–V* and symmetrical *I–V* characteristics ranging from −50 to 50 mV. Simultaneously, the device stabilities were investigated by characterizing conductance stabilities through 10^5^ reading operations, where the tiny standard deviations prove outstanding stability. In addition, the devices supply continuous‐time signals from the changing input voltage of the crossbar array, signifying potential for parallel computing. The experimental currents are consistent with the simulated values (Figure 2f). Moreover, systematic analysis has been conducted on the error statistics, considering variables such as random inputs, wire resistance, the dimensions of the crossbar array, and the spectrum of device resistance. This substantiates the high precision of frequency multiplexing computing (FMC)‐based parallel computing. Herein, the proposed FMC‐based parallel computing may supply a novel solution for parallel analog computers. Therefore, FMC‐based parallel computing may provide a new platform for low‐power intelligent edge applications, and solve real‐time processing and communication issues in the Internet of Things (IoT) networks. Li et al. showed a gate‐programmable van der Waals metal‐ferroelectric‐semiconductor vertical heterostructure memory, integrating ferroelectric and semiconductor functionalities to achieve multi‐level memory states with high on/off ratios and long‐term retention.^[^ ^78^ ^]^ The integration of ferroelectric and semiconductor materials in a vertical assembly provides a powerful platform for future nanoelectronic applications. On the other hand, traditional memory systems suffer from excessive data redundancy, additional hardware costs and increased power consumption, significantly hindering their performance. In contrast, 2D materials, with low cost and diverse engineering strategies, may be applied to develop new computing hardware and schemes, thereby realizing massively parallel computing and enhancing information processing speed in the future computing field.

**Figure 3:**
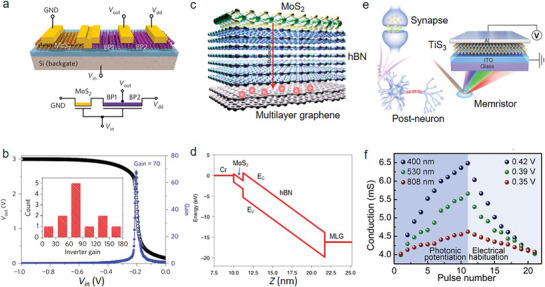
2D AM electronic applications. a) Schematic of the ternary inverter structure based on a MoS_2_ transistor (with varying channel length) in series with a BP channel. b) High gain of the inverter based on an in‐series BP FET (*L_ch_* = 5 µm) and MoS_2_ FET (*L_ch_* = 5 µm). The voltage gain can reach as high as 70 at *V_dd_* = 3 V. The inset figure shows the statistical distribution of voltage gains of multiple devices. Five of the devices exhibit a gain of ∼70, with a highest gain of over 150 (Reproduced with permission.^[^ ^79^ ^]^ Copyright 2017, Springer Nature). c) Schematic diagram of the MoS_2_/hBN/graphene van der Waals heterostructure. d) Band diagram of Cr/MoS_2_/hBN/MLG heterostructure from the technology computer‐aided design device simulation. Band diagram under the writing voltage *V*_*bg*,*pulse*_ = +30 V. *E_c_* and *E_v_* indicate the conduction and valence bands, respectively (Reproduced with permission.^[^ ^82^ ^]^ Copyright 2021, Springer Nature). e) Schematic diagram of optoelectronic artificial synapses based on 2D transition‐metal trichalcogenide. On the right side is a TiS_3_‐based memristor with the sandwich‐like Al/TiS_3_/ITO structure. f) Conductance change under optical and electric signals (Reproduced with permission.^[^ ^84^ ^]^ Copyright 2021, American Chemical Society).

Different band alignments of heterostructures determine unique electronic performance and transport properties. Huang et al. leveraged narrow‐bandgap and large‐bandgap MoS_2_ to form a vertical heterostructure with an ultrahigh rectification ratio (10^6^) and large on/off ratio (10^7^).^[^ ^79^ ^]^ This structure exhibits excellent performance with well‐rectification behavior due to the integration of diode and FET. Moreover, the ternary inverter has been designed based on MoS_2_ and BP (**Figure** **3**a). Specifically, this inverter contains three logic states: 1, 1/2 and 0. Since it is an in‐series circuit, every component carries an identical current and the circuitʼs voltage is the total of all components. At *V_in_* < −2.5 V, the inverter displays a high logic value of 1, which depends on the MoS_2_ʼs high resistance state. When −2.5 V < *V_in_* < −1.5 V, the MoS_2_ channel begins to activate, exhibiting greater conductance compared to other channels. It displays a mid‐range *V_out_* ≈ 0.55 V, corresponding to the partial voltage drops of both the BP2 FET and BP1 FET, as well as the MoS_2_ FET. As *V_in_* > −1.5 V, the MoS_2_ transistor is fully switched on. Both BP transistors are in the sub‐threshold region of the pFET, wherein the BP2 FET with a longer channel exhibits larger resistance than the BP1 FET, and consequently *V_out_* showcases a lower level of ≈0.1 V. Thus, we obtain three‐state‐output values that closely align with conventional logic values. For the ternary inverter, the intermediate state is controlled only by modulating the channel length. As two *I*_d_ *‐V*_g_ curves of the MoS_2_ and BP FETs intersect in the sub‐threshold areas by optimizing the channel length, a high‐voltage‐gain inverter was achieved as depicted in Figure 3b. An inverter featuring a channel length of 5 µm for both *L*_BP_ and $L_{MoS_{2}}$ demonstrates rapid switching capabilities and well‐aligned threshold voltages, when the input voltage shifts from 0 to −1 V. In addition, the gain reaches 31 at *V*_dd_ = 1 V and 70 at *V*_dd_ = 3 V. They also fabricated 12 BP‐MoS_2_ complementary‐metal‐oxide‐semiconductor (CMOS) inverters, and most exhibit high gains of >60. In particular, the highest gain can reach 152 when *V*_dd_ = 3 V, which is one of the highest gains in reported. Utama et al. reported the dielectric‐dependent bandgap renormalization and explored its influence on electrical transport.^[^ ^80^ ^]^ Domaretskiy et al. designed double ionic gated transistors to control the band structure of TMDCs.^[^ ^81^ ^]^ They demonstrated that the electronic band structure of 2D semiconductors was engineered through a perpendicular electric field, vertical heterostructure, and dielectric environment, leading to different device performance outcomes. Additionally, new physical phenomena, such as switching the magnetic anisotropy, the topological charge of magnetic excitations and control of the electronic state, etc., in the TMDCs, have been considered exclusively of academic interest in theoretical predictions.

2D vdW heterostructures also provide a powerful platform to improve the performance of memory. Liu et al. designed an ultrafast non‐volatile flash memory based on MoS_2_/hBN/multilayer graphene vdW heterostructures (MBG).^[^ ^82^ ^]^ The exceptional gate coupling ratio (GCR), minimal carrier tunneling barriers, and the atomically smooth interface of this design facilitate a writing speed on par with that of volatile random access memory (RAM), while still maintaining high density and non‐volatility. Figure 3c illustrates the schematic of a MBG heterostructure, in which bilayer MoS_2_, multilayer graphene (MLG) and hBN serve as the channel material, the floating gate layer and the tunneling layer, respectively. In addition, Liu et al. simulated the Cr/MoS_2_/hBN/MLG structure via computer‐aided design (Figure 3d), it was found that the MoS_2_/hBN layer has a large electric field and both layers illustrate a pronounced band slope. Specifically, the existence of two triangular barriers in the Cr/MoS_2_/hBN/layers was noted, with the barrier for MoS_2_/hBN being larger than that of Cr/MoS_2_. The barrier of MoS_2_/hBN determines the Fowler‐Nordheim (FN) tunneling current. However, these barriers are lower than the barrier (3–4 eV) of the Si/SiO_2_ interface, explaining the faster programming speed of MBG flash memory compared to its silicon counterparts. With an increase in the electric field, the energy band slope intensifies, and the Fermi energy of Cr exceeds the conduction band edge of hBN. Consequently, the high‐energy ʻlucky electronsʼ at Cr cross the small triangular potential barrier at the Cr/MoS_2_ interface without other potential barriers. These ‘lucky electronsʼ form a large tunneling current, leading to an ultrashort writing/erasing time. These results tally well with the theoretical calculations. Meanwhile, Wu et al. demonstrated that ultrahigh‐speed, non‐volatile, and floating‐gate memory devices with indium selenide (InSe)/hBN/MLG heterostructures attributed to interfacial coupling and atomically sharp interfaces.^[^ ^83^ ^]^ This device exhibits nanosecond read and write times, extremely high extinction ratio, and long‐retention time. The vdW heterostructure memory devices show uniform, clean and atomically sharp interfaces, different tunneling processes and various defects exhibit fast writing speed and ultra‐long retention time. They offer new operational schemes for electronic devices, holding great potential in commercial memory applications.

At the forefront of next‐generation computing, neuromorphic computing shines brightly, drawing inspiration from the ability of the human brain to process, store, and compute data simultaneously. Liu et al. have made significant strides in this field by designing an optoelectronic memristor using a 2D transition‐metal trichalcogenide (TMTC), specifically showcasing stable bipolar resistance switching (RS) as seen in Figure 3e.^[^ ^84^ ^]^ The memristor realized various synaptic functions including conduction modulation, photonic potentiation and spiking timing‐dependent plasticity (STDP), effectively mimicking Pavlovian associative learning through a TiS_3_‐based artificial synapse. This optoelectronic memristor that duplicates visual sense reception is simultaneously controlled by optical signals and electricity. Specifically, the memristor contains Al/TiS_3_/Indium tin oxide (ITO). Under light illuminations, plenty of photoinduced carriers generate in TiS_3_, leading to the migrations and accumulations of excessive electrons around Al filaments, which are crucial for the RS characteristics observed in the device. Figure 3f illustrates that light‐induced potentiation alongside electric‐induced habituation can modulate the conductance and synaptic weight of the TiS_3_‐based memristor. Itʼs noted that the conductance enhancements vary with different wavelengths, increasing to 6.36, 5.51, and 4.55 mS under different illumination. The study highlights that short wavelengths are particularly effective in generating photogenerated carriers, thereby increasing conductance significantly. Besides that, this conductance change remained over 100 s after switching off the light. These results demonstrate TMTC‐based memristors offer tremendous potential in neuromorphic computing applications. Syed et al. reported a type‐II, 2D heterostructure‐based optomemristive neuron using MoS_2_, WS_2_ and graphene, demonstrating both excitatory and inhibitory effects via optoelectronic charge‐trapping mechanisms.^[^ ^85^ ^]^ The device is applied in simulations for unsupervised competitive learning (CL) and cooperative learning (CoL) tasks, suggesting its potential in machine learning and neuromorphic computing. The 2D material system provides a framework for future developments in electron devices and their integration into advanced computing systems. In summary, 2D materials exhibit ideal traits for electronic devices with various nanostructures: heterostructures, superlattice structure, 2D material ribbons, crossbar array, solid‐state ionic doping, plasmonic antennae based on 2D material, and so on. These nanostructures cover several main applications, such as valleytronic devices, superconductive devices, storage, and computing devices. Therefore, the development of 2D material‐based nanostructures is an important candidate for the realization of mass‐manufactured and widely used electronic devices in the future. Meanwhile, the economical and uniform production of defect‐free 2D materials, high‐quality heterostructures or superlattice structures that have no wrinkling, folding, tearing, and cracking ribbons, or tube‐like and unique patterning‐based 2D material should be further pursued.

## 3 Photonic Devices

**Figure 4:**
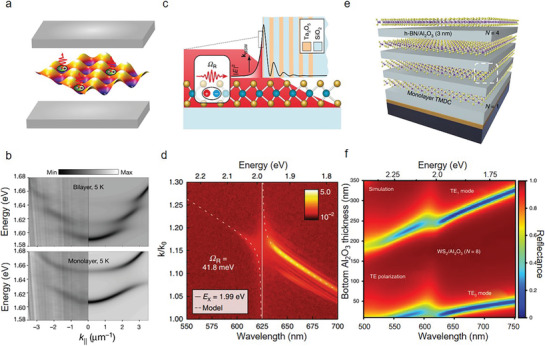
2D AM photonic applications. a) Schematic of the moiré polariton system, formed by excitons confined in a moiré lattice and coupled with a planar microcavity. WS_2_‐MoSe_2_ hetero‐bilayers are enclosed in the microcavity. b) Bilayer (top) and monolayer (below) angle‐resolved white‐light reflection spectra at 5 K. The left and right panels of each image show the measured and simulated results (Reproduced with permission.^[^ ^97^ ^]^ Copyright 2021, Springer Nature). c) Schematic of the dielectric stack supporting BSW polaritons in monolayer WS_2_. Black curve shows the electric field profile of the bare mode (TE‐polarized) at the wavelength of the A exciton band. The inset illustrates the coupling of the enhanced electric field at the surface of the stack to the in‐plane excitons in the monolayer. d) Experimental dispersion in photoluminescence under 514 nm excitation on a logarithmic colour scale. The measurement and simulation results are represented by dotted lines and solid lines respectively. The anti‐crossing of exciton energy near 1.99 eV/623 nm indicates a strong coupling state (Reproduced with permission.^[^ ^98^ ^]^ Copyright 2018, Springer Nature). e) An all‐vdW superlattice composed of alternating layers of TMDC and hBN, and a mixed dimensional superlattice with alternating layers of TMDC and 3D insulating oxides grown via ALD. *N* = 1 and *N* = 4 represent the unit cell number respectively. f) In the WS_2_/Al_2_O_3_ stack with *N* = 8, the bottom alumina thickness and wavelength determine the reflectance with the TE_0_ and TE_1_ modes labelled (Reproduced with permission.^[^ ^106^ ^]^ Copyright 2022, Springer Nature).

The collective coupling of quantum particle arrays manipulates light‐matter interactions, having great potential for fabricating atomic optical lattices and cavities. **Table** **1** provides detailed key metrics of 2D AM lasers, illustrating strong exciton‐photon interactions. The structures encompass dielectric microcavities, Fabry‐Perot cavities, microdisk resonators and photonic crystal cavities.^[^ ^86^, ^87^, ^88^, ^89^, ^90^, ^91^, ^92^, ^93^, ^94^, ^95^, ^96^ ^]^ These designs enhance light‐matter interactions and reduce the lasing threshold of the emitter. The cavity‐gain coupling in both the surface‐gain geometry and the embedded quantum‐dot structure can achieve ultralow thresholds by enhancing spontaneous emission into a resonant cavity mode. Notably, embedding quantum dots into a photonic crystal cavity separates the nanocavity from gain materials. This approach facilitates the individual fabrication of both parts at high quality before their non‐destructive and deterministic combination as hybrids. These results contribute to realistic applications in a scalable and designable manner, compatible with integrated electronic circuits. It is well accepted that the moiré lattice is induced by the tiny mismatch in their lattice constants or crystal orientations of the two monolayer (ML) crystals forms, enabling correlated electron gases within the lattice. Zhang et al. established cooperative coupling between moiré‐lattice excitons and photons by integrating MoSe_2_/WS_2_ hetero‐bilayers in a microcavity (**Figure** **4**a).^[^ ^97^ ^]^ These hetero‐bilayers, encapsulated by hBN, were fabricated by a twist angle of 56.5° ± 0.9°. The two low‐energy exciton modes within the moiré lattice showed substantial oscillator strengths, inherited from the A‐exciton of ML MoSe_2_. This feature not only aids in the detection of moiré excitons through their absorption spectra but also indicates a profound interaction between moiré excitons and photons, leading to the formation of stable moiré polaritons. Angle‐resolved white‐light reflection spectra exposed anti‐crossing modes at the moiré exciton resonance points, illustrating the intense coupling between moiré excitons and photons, as shown in Figure 4b. The observed variability in moiré polaritonsʼ density indicates to pronounced nonlinearity, manifesting from exciton blockade, diminished shifts in exciton energy, and reduced dephasing caused by excitation, aligning with the quantum‐confined nature of moiré excitons. The moiré polariton framework, marked by its significant nonlinearity and capability for microscopic‐scale adjustment of matter excitations, serves as a promising platform for exploring collective behaviors through adjustable quantum emitter arrays.

**Table 1:** Strong exciton‐photon interaction in 2D AMs and the key metrics of 2D AMs lasers.

Materials	Structure	Temperature [K]	Rabi splitting [meV]	Wavelength [nm]	Linewidth	Pump	Threshold	Refs.
MoS_2_	Dielectric microcavity	Room temperature (RT)	46 ± 3	–	–	–	–	[86]
MoSe_2_	–	RT	100	383	–	–	–	[87]
WS_2_	Fabry‐Perot cavity	RT	70	Far below the stopband of the mirror	60 meV	–	–	[88]
WSe_2_	Fabry‐Perot cavity	RT	23.5	580–780	35 meV	–	–	[89]
MoSe_2_/hBN	Tunable microcavities	4.2	29	750	1.635 meV	–	–	[90]
WS_2_	Microdisk resonator	10	–	612	0.24nm	473 nm, femtosecond pulse	5–8 MW cm^−2^	[91]
WSe_2_	Photonic crystal cavity	130	–	740	≈0.6 nm	632 nm, continuous wave	27 Nw (1 W cm^−2^)	[92]
MoTe_2_	Silicon photonic‐crystal cavity	RT	–	1132	0.202 nm	633 nm, continuous wave	6.6 W cm^−2^	[93]
MoS_2_	Silica microsphere cavities	77–400	–	600–800	0.3–2.05 nm	532 nm, continuous wave	32–580 W cm^−2^	[94]
BP	SiO2/Si3N4 open microcavity on silicon	RT	–	≈3765	9 nm	740 nm, femtosecond pulse	850 mW	[95]
WSe_2_/MoS_2_	Nanocavity	RT	–	1128	2.26nm	740 nm, continuous wave	7 kW cm^−2^	[96]

Moreover, microcavity exciton‐polaritons emerge when the interaction between excitons and cavity photons surpasses their separate rates of decay, leading to strongly polariton interactions and valuable nonlinear phenomena. TMDCs can overcome the limitations of cryogenic temperatures and weak nonlinearities, showing strong light‐matter coupling further contributing to room‐temperature polariton devices. Meanwhile, the Bloch surface wave has emerged as a superior platform for the manipulation of polariton fluids within monolayer TMDCs, confining the electric field to a diminutive volume adjacent to the dielectric mirror surface. Barachati et al. designed a nonlinear polariton source based on monolayer WS_2_ (Figure 4c). They coated the dielectric mirror on the glass‐slip cover and then transferred a large monolayer of WS_2_ onto the dielectric mirror. The experimental results illustrate that the mode is TE‐polarized and travels along the surface, characterized by the wavevector *k_BSW_* (Bloch surface wave), highlighting an improved electric field at the surface and exciton couplings in the monolayer. In addition, the photoluminescence spectra show that there is an anti‐cross phenomenon at 1.99 eV (Figure 4d), suggesting strong couplings. The observed Rabi splitting align close to the calculated transfer matrix values. Monolayer WS_2_ sustains Bloch surface wave polaritons (BSWPs) with a Rabi splitting of 43 meV and propagation lengths of ≈33 µm.^[^ ^98^ ^]^ These results herald new possibilities for integrated optical processing and the development of polaritonic circuits. For vdW heterostructures, the elegant interaction of charge, spin and moiré superlattice structure with many‐body effects triggers novel and rich exciton phenomena as well as quantum phase transitions.^[^ ^99^, ^100^, ^101^, ^102^, ^103^, ^104^ ^]^ Sun et al. reported a transition in the nature of dipolar interaction among interlayer excitons from repulsive to attractive in the freestanding twisted WSe_2_/WS_2_ bilayers.^[^ ^99^ ^]^ These suspended heterostructures show enhanced many‐body interactions, leading to high densities of interlayer excitons and strongly enhanced dipole‐dipole interactions, which induce the quantum‐exchange correlation phases and robust interlayer biexcitons. Additionally, Zhang et al. employed mono/few‐layer organic single crystals to fabricate 2D organic‐inorganic heterostructures that show efficient and layer‐dependent energy transfer.^[^ ^105^ ^]^ This phenomenon is attributed to the layer‐dependent carrier transport properties and high quantum yields of organic counterparts. Furthermore, Zhang et al. observed novel Type II interlayer trions in the 1L WSe_2_/2L pentacene heterostructure by designing suitable band alignments, high free charges, high coulomb interaction and high interface quality.^[^ ^59^ ^]^ These findings promote the design and development of exciton devices and all‐optical circuits.

Traditional Semiconducting multi‐quantum wells (MQWs) and superlattices are fundamental to the development of high‐efficiency optoelectronic and photonic devices. However, their complicated growth process hampers absorption and applicability. VdW semiconductors provide a solution by transferring methods. Despite large refractive indices and exciton binding energy, the interactions between vdW semiconductors and light are weak. Thus, it is necessary to construct superlattices by repeating units in one unit. Kumar et al. reported a square‐centimeter scale and multilayer superlattice structure capable of achieving nearly 90% light trapping at excitons in an active‐layer absorber of <4 nm.^[^ ^106^ ^]^ Figure 4e shows the schematic of the multilayer superlattice. When light is coupled into the superlattices at an incidence angle >45°, the strong‐coupling excitonic‐polariton occurs. It adjusts the exciton‐polariton dispersion and coupling strength through altering the superlattice's geometric parameters and unit cells. In the WS_2_/Al_2_O_3_ heterostructure, when *N* = 8 (stack number) with TE_0_ and TE_1_ modes labeled, the reflectivity depends on the thickness and wavelength of the bottom alumina (Figure 4e). Transfer Matrix Method (TMM) simulations suggest that excitons hybridize with cavity modes within the superlattice, giving rise to exciton polaritons. The energy of the Rabi splitting associated with these polaritons is determined by the angle of incidence and the number of unit cells in the structure (Figure 4f). The appearance of anti‐crossing behavior indicates the successful formation of polaritons, a phenomenon that can be further confirmed by adjusting the cavity resonance through alterations in the thickness of the bottom alumina layer. As reported, the Rabi splitting increases to 186 meV when *N* = 8 and the incident angles >80°. These results pave the way to design large‐scale optical metamaterials based on atomically thin layers.

**Figure 5:**
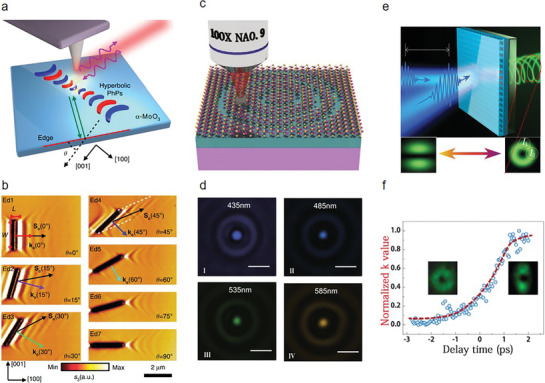
2D AM photonic applications. a) Schematic diagram of edge‐tailored PhPs in *α*‐MoO_3_. Green arrows indicate the incident PhPs waves launched by the laser‐illuminated (purple curve arrows) AFM tip and reflected by the edge (red line). b) Real‐space imaging of edge‐tailoring PhPs at angle‐dependent *α*‐MoO_3_ edges (width W: 200 nm; length L: 2.5 µm; sample thickness d: 210 nm, L and W defined in the Ed1) (Reproduced with permission.^[^ ^107^ ^]^ Copyright 2020, Springer Nature). c) Illustration of a binary phase‐type SCL on a monolayer MoS_2_ sheet obtained by fs laser scribing. d) Experimentally measured intensity profile of an atomically thin SCL at the focal plane for selected wavelengths (Reproduced with permission.^[^ ^108^ ^]^ Copyright 2021, Springer Nature). e) Schematic of the two‐beam pumping experiment. The bottom shows the far‐field emission patterns from the perovskite metasurface under both symmetric and asymmetric excitations. f) Transition from a BIC microlaser to a linearly polarized laser. Insets show the corresponding beam profiles (Reproduced with permission.^[^ ^109^ ^]^ Copyright 2020, The American Association for the Advancement of Science).

Polar vdW materials have in‐plane hyperbolic phonon polaritons (PhPs), providing a nontrivial platform for optical manipulations at the nanoscale. In addition, nanocavities or nanoresonators that consist of polar vdW materials enhance wave confinements and light‐matter interactions. Dai et al. manipulated and directed in‐plane hyperbolic PhPs via tailoring the edge orientations of a resonant nanocavity in biaxial *α*‐MoO_3_ (**Figure** **5**a).^[^ ^107^ ^]^ Utilizing nanoscale Fourier‐transform infrared spectroscopy, the researchers have shown that isosceles triangle and rectangular α‐MoO_3_ nanocavities can support edge‐tailored PhPs with in‐plane anisotropic propagations. Figure 5b illustrates a series of grooves with varying edge orientation angles that were fabricated. As *θ* increases from 0° to 45°, interference fringes that are parallel to the edge lines continue to be present, albeit with a growing angle between the propagation direction and the wavevector. Notably, it is not observed that no PhP fringes parallel to the edges occur when *θ* ≥ 60°. The relationship between the edge angle and the crystallographic direction critically affects the distribution of PhPs, thereby substantially altering the optical response of the system. This breakthrough underscores the potential of polar vdW materials in facilitating advanced optical functionalities through nanoscale engineering.

Monolayer TMDCs can modify wavefronts with ultrahigh spatial uniformity. To control the phase modulations precisely, Qin et al. introduced the loss‐assisted singular phase behavior near the critical coupling point by depositing monolayer MoS_2_ on a ZnO/Si substrate.^[^ ^108^ ^]^ The ZnO layer displays an increased refractive index and transparency within the visible light spectrum, combined with the infinite thickness of the Si layer preventing any light transmission, forming the basis of a supercritical lens (SCL). This lens, crafted using a direct femtosecond laser scribing technique, is capable of achieving sub‐diffraction‐limited focusing and imaging in the far field by modulating interference conditions from each zone belt that supports different spatial components, as illustrated in Figure 5c. The focal spots of SCL keep their lateral size at 640 ± 50 nm from 435 to 585 nm, showing a strongly dispersive spot size (Figure 5d). More interestingly, the broadband phase shift is significantly expanded through the thickness of MoS_2_. A large phase shift of over π/2 was obtained in bilayer MoS_2_ sheets within the 435–585 nm range, affirming the mechanism of phase shift assisted by loss. The SCL with π phase modulation reduces the optical element to the atomic thickness, enabling a π phase jump. This creates a new way for self‐modulating photoluminescence, exciton FET, function‐integrated photodetection and next‐generation optical computing.

The advancement in both classical and quantum information processing technologies has spurred a demand for high‐performance integrated sources of structured light. Vortex beams, distinguished by their varying topological charges and mutual orthogonality emerge as a promising solution. Currently, reported integrated vortex microlasers are hindered by static designs and relatively high thresholds required for lasing, rendering them less effective for rapid optical communication and computing applications. Huang et al. designed perovskite‐based vortex microlasers and realized an ultrafast all‐optical switching at room temperature.^[^ ^109^ ^]^ By leveraging bounded states in the continuum (BICs), which emerge from the interference between localized resonances and radiation modes, they have managed to achieve both high modulation speeds and low energy consumption. This innovation demonstrates that BIC vortex microlasers maintain their robustness across more than ten samples, indicating a significant reliability in their performance. As depicted in Figure 5e, a circular laser beam is used to pump the perovskite metasurface, maintaining it's symmetry to produce a donut‐shaped beam. However, modifying the shape of the excitation area from circular to elliptical breaks this symmetry, resulting in the generation of two linearly diffracted beams. Moreover, employing a double‐pump configuration with a specific time delay offers insights into the temporal dynamics of the transition process, as shown in Figure 5f. Remarkably, the transition from a vortex beam laser to a linearly polarized laser occurs in ≈1.5 ps, due to the far‐field nature of BICs. This rate is significantly faster than previously reported technologies, with the transition times for switching between two linearly polarized beams to vortex microlasers being comparably swift. The energy efficiency of these BIC vortex lasers is notably superior to that of traditional all‐optical switches, presenting a considerable advantage for future applications in optical computing and communication. In conclusion, 2D materials offer promising solutions for a wide range of optical applications, including photodetectors, modulators, lasers and sensors. These solutions are based on their broad spectrum, high carrier mobility, tunable bandgaps, strong light‐matter interactions, high optical gain, large surface‐to‐volume ratio and high sensitivity. While the research on 2D materials in optical devices has made impressive strides, addressing some challenges is vital for transitioning from laboratory‐scale demonstrations to commercial applications, such as scalable production techniques, environmental stability and interface engineering.

## 4 Optoelectronic Devices

**Figure 6:**
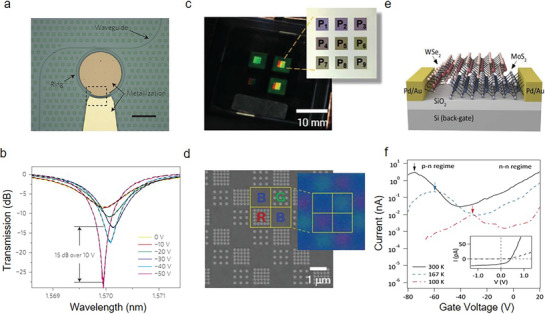
2D AM optoelectronic applications. a) Optical micrograph of the proposed graphene electro‐optic modulator. b) The measured and calculated curves for variable applied direct current voltages on the designed structure (Reproduced with permission.^[^ ^112^ ^]^ Copyright 2015, Springer Nature). c) Photograph of a meta‐OLED test cell. The inset displays an optical microscopy picture of 3×3 metamirrors with various pitches. d) SEM image with 1.2 µm subpixel pattern and accompanying EL image. A BGRB pixel is shown by the yellow box (Reproduced with permission.^[^ ^137^ ^]^ Copyright 2020, The American Association for the Advancement of Science). e) Schematic illustration of the MoS_2_/WSe_2_ heterostructure solar cell. f) Temperature dependent device current, recorded by scanning the back‐gate voltage *V_G_*. Larger positive *V_G_* selection shows resistive n‐n behavior. For proper *V_G_* selection, an atomically thin p‐n junction is formed, which displays a rectification behavior similar to that of a diode. The arrows in the figure point to the p‐n state. Inset: current‐voltage characteristic at room‐temperature, recorded at *V_G_* = −75 V. Dashed line: dark current; solid line: under optical illumination (Reproduced with permission.^[^ ^138^ ^]^ Copyright 2018, Springer Nature).

High‐performance 2D AM‐based optoelectronic devices, including artificial synapse,^[^ ^110^ ^]^ photovoltaic devices,^[^ ^111^ ^]^ electro‐optic modulators,^[^ ^112^ ^]^ photonic microring‐photodetectors,^[^ ^113^ ^]^ waveguide photodetectors,^[^ ^114^ ^]^ image arrays,^[^ ^115^ ^]^ and metasurface organic light‐emitting diode (OLED), etc., have been heavily pursued.^[^ ^113^, ^114^, ^116^, ^117^ ^]^ The key metrics of photodetectors are summarized in **Table** **2**. Their responsivities span from 10^−6^–10^6^ A W^−1^.^[^ ^118^, ^119^, ^120^, ^121^, ^122^, ^123^, ^124^, ^125^, ^126^, ^127^, ^128^, ^129^, ^130^, ^131^, ^132^, ^133^, ^134^, ^135^ ^]^ Some photodetectors reach sub‐millisecond response times and high detectivity of >10^9^ Jones,^[^ ^123^, ^124^, ^127^, ^130^ ^]^ operating across a broad spectral range. For instance, atomically thin graphene/WS₂ heterostructure photodetectors that were encapsulated in an ionic polymer can operate at bandwidths up to 1.5 kHz while maintaining sub‐millisecond response times and a responsivity *R* of 10^6^ A W^−1^.^[^ ^121^ ^]^ Ionic polymers can compensate for charge traps, thereby circumventing their limitation on responsivity and response time. The combination of high responsivity and fast response renders these photodetectors suitable for video‐frame‐rate imaging applications. MoS_2_/graphene/WSe_2_ heterostructure photodetectors that were formed by sandwiching graphene in an atomically thin p‐n junction, enable broadband photodetection in the visible to short‐wavelength infrared range.^[^ ^124^ ^]^ They show competitive device performance, including a specific detectivity of up to 10^11^ Jones in the near‐infrared region. Recently, Phare et al. designed a graphene electro‐optic modulator by integrating graphene atop a ring resonator (**Figure** **6**a).^[^ ^112^ ^]^ This new modulator demonstrates super‐low losses, extensive transparency range, and an impressive modulation efficiency reaching 15 dB for every 10 V, as shown in Figure 6b. When examining the device's transmission with varying gate voltages, a transformation in the cavity's line shape was observed. Initially, at 0 V, it showed a resonance characteristic of low quality (Q) and under‐coupling, which transitioned to a resonance of higher Q and coupling as the voltage increased. Peak transmission is attributed to the unique, non‐linear gate‐dependent conductivity of graphene. Similar results happen in devices when graphene is integrated into photonic crystal cavities. Xu et al. demonstrated a stable and magnetically tunable deep‐ultraviolet birefringent optical modulator using 2D hBN, achieving significant birefringence effects and high stability for deep‐ultraviolet (DUV).^136^ ^]^ The large specific Cotton‐Mouton coefficient and ultrawide bandgap of hBN enable efficient and practical DUV light modulation, making it suitable for a wide range of technological applications. Joo et al. devised a new OLED structure based on spatially varying metaphotonic Fabry‐Pérot (FP) cavities.^[^ ^137^ ^]^ The emission spectra of designed meta‐OLED pixels can be significantly modulated across the whole visible spectrum by precisely designing the nanopatterns on Ag reflectors (Figure 6c). By utilizing the optimized microcavity effects, a clear increase in the aspects between color purity and luminescence efficiency can be obtained. The OLED structure contains red, green, and blue (RGB) emitters and charge‐transport layers atop mirrors. This cost‐effective fabrication strategy for RGB pixels offers scalability to the square‐meter scale that is pivotal to implementing meta‐OLED technology. Moreover, the RGB subpixels show a decreased size from mobile display levels to *µ*‐displays. The RGB colors from large subpixels to sub‐pixels substantiate the phase modifications of metamirrors (Figure 6d). They do not exhibit any color distortions or luminescence reductions. The advancement heralds a significant leap toward creating efficient RGB pixels for OLEDs, eliminating the need for fine‐metal masks and color filters. 2D materials have garnered significant attention in the field of optoelectronics, due to their unique electronic and optical properties, atomic‐scale thickness and high surface area‐to‐volume ratio. Additionally, the distinctive properties of 2D materials enable new functionalities and improved performance, paving the way for next‐generation optoelectronic devices. Furthermore, continued research and development in areas such as material quality and scalability, interface engineering, integration and packaging will address current challenges and unlock further potential in this exciting area.

**Table 2:** The key metrics of photodetectors.

Structure	Responsivity [A W^−1^]	Response time [s]	Detectivity [Jones]	Wavelength [nm]	Refs.
MoS_2_	8.45 × 10^−6^	0.976	4.1 × 10^7^	600–700	[118]
WSe_2_	2.2 × 10^6^	0.3	1.6 × 10^13^	–	[119]
Graphene	–	–	–	–	[120]
WS_2_‐Graphene	10^6^	1.3 × 10^−4^	3.8 × 10^11^	400–700	[121]
MoS_2_‐Graphene	0.835	0.02	–	–	[122]
Graphene−Black Phosphorus	3.3 × 10^3^	4 × 10^−3^	1.13 × 10^9^	400–1600	[123]
MoS_2_−Graphene−WSe_2_	10^4^	5.36 × 10^−5^	10^11^	400–2400	[124]
Graphene‐WSe_2_‐Graphene	1.2 × 10^−4^	1.3 × 10^−12^	–	–	[125]
PTCDA‐Pentacene	10^5^	2.8 × 10^−5^	–	400–700	[126]
Graphene/GaAs	0.21	–	2.98 × 10^13^	325–980	[127]
Graphene/Si	0.2	5 × 10^−9^	1.6 × 10^13^	200–400	[128]
PEA_2_FA_n‐1_Pb_n_X_3n+1_	1.38 × 10^−2^	–	10^11^	500–900	[129]
PbS/MoS_2_	10^6^	0.3	10^11^	800–1100	[130]
Nb/MoS_2_	4.83 × 10^5^	4.9 × 10^−3^	5.0 × 10^12^	450	[131]
MoS_2_ /hBN/graphene	180	0.23	2.6 × 10^13^	532	[132]
SnS_2_‐QDs/MoS_2_	278	0.1	4.589 × 10^12^	365	[133]
SnS_2_‐QDs/MoS_2_	435	0.1	7.19 × 10^12^	554	[133]
SnS_2_‐QDs/MoS_2_	189	0.1	3.11 × 10^12^	780	[133]
PdSe_2_/MoS_2_	42.1	7.45 × 10^−2^	8.21 × 10^9^	10.6	[134]
TiS_3_/MoS_2_	48 666	0.4	10^14^	365–940	[135]

2D semiconductors are also applied into photovoltaic devices, due to the high internal radiation efficiency, strong optical absorption, and tunable band gaps, etc. Currently, 2D material‐based photovoltaic devices have been realized in lateral p‐n junctions, vertical heterostructures, homojunctions and nanotubes. Furchi et al. investigated the carrier transport mechanism in type II MoS_2_/WSe_2_ heterostructures by developing a model that reproduces the current‐voltage characteristics under optical illuminations.^[^ ^138^ ^]^ As illustrated in Figure 6e, the configuration of the solar cell heterostructure includes a MoS_2_ layer serving as the electron transport layer (ETL) and WSe_2_ as the hole transport layer (HTL), estimating an effective band gap (*E_g,eff_*) which is the difference between the ETL and HTL energy levels, roughly calculated as *E_(CB,M)_* – *E_(VB,W)_*, equaling ≈1.3 eV. When a suitable gate voltage (*V_G_*) is applied, it shows an atomically thin p‐n junction and diode‐like behavior in its *I*–*V* characteristics. The larger positive V demonstrates the resistive n‐n behavior (Figure 6f). Through experimental observations, the external quantum efficiency (EQE) was ≈1% under a low light intensity of ≈1 kW m^−2^ at a 532 nm wavelength, which diminishes as light intensity escalates. Remarkably, the highest EQE observed for the MoS_2_/WSe_2_ heterostructure surpasses 50%, with an absorbance rate of over 90% and a power conversion efficiency (PCE) of 3.4%. Zhang et al. demonstrated a WS_2_ nanotube with reduced crystal symmetry extending beyond mere broken inversion symmetry, which transitioned from a 2D monolayer to a nanotube with polar properties.^[^ ^139^ ^]^ Reduced crystal symmetry provides importance for enhanced bulk photovoltaic effect (BPVE) and converting solar to electric power. Furthermore, edge‐embedded vdW structures are fabricated with strong symmetry‐breaking, low dimensionality, and abundant species,^[^ ^140^ ^]^ and the structures support a strong BPVE under polarized light, leading to a measurable photocurrent flowing in the y‐direction. Bulk photovoltaic effects have emerged in vdW nanostructures, superlattices, Reyl semimetals, bulk Rashba semiconductors, ferroelectric insulators, and so on, which further contribute to improving the efficiency of energy harvesting. Furthermore, the integration of 2D materials into PV devices signifies a substantial advancement in solar energy technology. These materials possess unique properties that augment the efficiency and stability of PV devices. Continued research will contribute to unlocking the full potential of 2D materials in solar energy applications.

**Figure 7:**
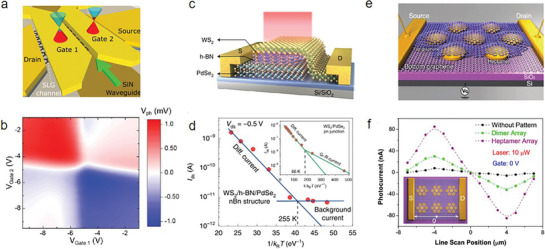
2D AM photodetectors. a) Scheme of waveguide‐integrated, plasmonic enhanced graphene photodetectors. SLG on SiN waveguide (brown) with split‐gates, acting as plasmonic slot waveguide, to create a p‐n junction in the channel. The green arrow indicates the light propagation direction. b) Photovoltage map for zero bias (Reproduced with permission.^[^ ^141^ ^]^ Copyright 2019, American Chemical Society). c) Schematic illustration of the WS_2_/hBN/PdSe_2_ vdW unipolar barrier photodetector. d) Arrhenius plot of the current of the nBn vdW unipolar barrier device exhibiting a high‐operating‐temperature characteristic. The inset figure shows Arrhenius plot of the dark current of the PdSe_2_/WS_2_ junction at a bias of −0.5 V (Reproduced with permission.^[^ ^144^ ^]^ Copyright 2021, Springer Nature). e) Schematic diagram of graphene‐antenna sandwich photodetector. A gold heptamer is sandwiched between two monolayer graphene sheets. f) Photocurrent measurements show antisymmetric photocurrent responses from the different regions of the device corresponding to specific plasmonic antenna geometries. The black dots indicate the photocurrent detected without the plasmonic antennas present in the device. The zero point is indicated in the inset schematic (Reproduced with permission.^[^ ^147^ ^]^ Copyright 2012, American Chemical Society).

To respond to growing global data, it is imminent to exploit an efficient and low‐cost optical communication system. Traditional Si photonics‐based receivers (Ge or bonded III−V PDs) can enable dense integrations. Nevertheless, defects and threading dislocations within Ge epilayers and at interfaces lead to an undesirable dark current, hindering an improvement in the operation speed without compromising responsivity (*R_I_*). Muench et al. proposed a micrometer‐scale graphene photodetector (GPD) for telecom wavelengths operating at zero dark currents, showing an external responsivity of ≈12.2 V W^−1^, a bandwidth of 3 dB and high‐speed operation of ≈42 GHz.^[^ ^141^ ^]^ The plasmonic‐enhanced photodetector is comprised of two gold gates placed above the graphene channel, which are insulated from the single‐layer graphene (SLG) via the Al_2_O_3_ and precisely aligned along the waveguide's center, resulting in a split gate function, as shown in **Figure** **7**a. Electrostatic split gates create a p‐n structure within the SLG channel, guiding a restricted surface plasmon polariton (SPP) waveguide mode. As the guided signal on the chip reaches the photodetector area, it undergoes an evanescent transition from the silicon nitride (SiN) waveguide into the split gate, serving as an SPP waveguide. By employing strategies that capitalize on optical field enhancement and plasmonic confinement within the gap, significant augmentation between light and SLG as well as enhanced optical absorption within the p‐n structure region is achieved. This approach leads to the creation of a localized heat source from confined electrons, contributing to a more compact device architecture. As illustrated in Figure 7b, the device exhibits a heightened response in bipolar (p‐n, n‐p) structure, contrasted with a diminished response in unipolar (n‐n, p‐p) structure, marked by a faint diagonal sign crossover when *V*_*Gate*1_ = *V*_*Gate*2_. When the GPD is at zero drain‐source voltage, the photodetection mechanism is predominantly dominated by the photo‐thermoelectric effect, with dual sign shifts in *V_ph_* during a singular gate sweep, indicative of the Seebeck gradient across the junction, due to Seebeck's non‐monotonic‐dependence‐on‐carrier‐mobility. Furthermore, Koepfli et al. demonstrated a zero‐bias, graphene‐based photodetector with >500 GHz electro‐optic bandwidth. In particular, these devices show a 200‐nanometer‐wide spectral band with a center wavelength from ≈1400 to ≈4200 nm.^[^ ^142^ ^]^ The combination of speed, bandwidth, and spectral versatility highlights the technological superiority of GPDs over existing photodetector technologies, which opens new avenues for innovation in optical communication systems and advanced sensing solutions. Pang et al. reported a non‐volatile rippled‐assisted optoelectronic array for all‐day motion detection and recognition, integrating sensing, memory and computation in a single device inspired by snake vision systems.^[^ ^143^ ^]^ The study establishes a model platform for integrating future intelligent optoelectronic devices, advancing the field of motion detection and recognition technology. The advancements in optoelectronic communication devices represent significant progress in photonics and electronic devices, paving the way for next‐generation communication technologies.

The unique property of 2D materials without surface dangling bonds enables unprecedented capabilities to stack vdW heterostructures, which facilitate the fabrication of unipolar barrier photodetectors. Comparatively, group III‐Vtransition metals and HgCdTe, etc., have lattice mismatch and interface defects for heterostructures. Chen et al. reported unipolar barrier photodetectors based on vdW heterostructures, which can block dark currents without suppressing the photocurrent due to passivated surfaces.^[^ ^144^ ^]^ Specifically, they proposed two unipolar‐barrier photodetectors: the nBn photodetector (WS_2_/hBN/PdSe_2_ heterostructure) and the pBp photodetector (BP/MoS_2_/graphene heterostructure). In the nBn photodetector, WS_2_ functions as the light‐absorbing layer, hBN serves as the barrier layer, and PdSe_2_ acts as the contact layer, respectively (Figure 7c). This design enables the device to operate effectively under reverse bias: photogenerated electrons from the n‐type absorber are efficiently collected at the anode, while the photoinduced holes in the WS_2_ layer are directed toward the cathode through a minimal valence‐band offset (*ΔE_v_*). However, the electrons within the PdSe_2_ layer are prevented due to a significant barrier in the conduction band. The performance of these devices, especially their ability to suppress dark currents, was further illustrated through the Arrhenius plot presented in Figure 7d, which displays the behavior of dark currents as a function of temperature at a fixed voltage of −0.5 V for both the nBn and junction structures. Remarkably, the dark current levels remain constant below 255 K, indicating the high‐temperature suitability of these devices. Li et al. demonstrated a reconfigurable and non‐volatile neuromorphic photovoltaic device based on a metal‐semiconductor‐metal (MSM) structure using 2D metal sulfides (MoS₂ and WS₂). They achieved tunable photocurrent responses through the modulation of sulfur vacancies, enabling flexible and stable operation.^[^ ^145^ ^]^ The neuromorphic photovoltaic device exhibits capabilities for image processing, object detection and mimicking neurobiological functions. Additionally, Song et al. demonstrated that fluoride substrates (e.g., CaF_2_) with (111) surface offer quasi‐vdW devices with comparable performance improvement as hBN and can be made at wafer‐scale with direct deposition approaches.^[^ ^146^ ^]^ The quasi‐vdW structures of SnS_2_/CaF_2_ and WS_2_/CaF_2_ exhibit one order of higher carrier mobility and photoelectric response rate than SnS_2_/SiO_2_ and WS_2_/SiO_2_. The advancements in barrier devices based on 2D materials signify important progress in optoelectronics. Continued research and development will further explore their applications to sensors, flexible electronics and quantum devices.

Although graphene's exceptional properties, such as high electron mobility and remarkable mechanical flexibility make it a highly sought‐after material for various applications, the potential in optoelectronic applications has been limited due to its inherently low absorption cross‐section and quantum efficiency. Integrating graphene with noble metal films or nanoparticles mitigate the above shortcomings through surface‐plasmon effects. For example, Fang et al. designed a graphene‐antenna‐sandwich photodetector, effectively converting visible light and near‐infrared photons into electrons (Figure 7e).^[^ ^147^ ^]^ Herein, the plasmonic cluster (composed of a transparency window) inherits the merits of nanoscale antennas and graphene, exhibiting tunable Fano resonances. The unique design significantly suppresses scattering within the plasmonic cluster, with the primary mechanism of absorption being the generation of electron‐hole pairs, closely tied to the transparency window's near‐field effects. When incident light resonates with the transparency window, it significantly amplifies the near‐field intensity, thereby boosting the direct excitations (DE) of electron‐hole pairs in the modified MG. In addition, zero‐bandgap graphene allows hot electrons (HE) generated within the gold structure and then transferred to its conduction band. Due to the graphene's high mobility, these excited charge carriers, both DE and HE directly move into the electrical circuit and form photocurrents. An antisymmetric photocurrent response, highlighted by Figure 7f, is observed when scanning with a 785 nm excitation laser, where regions with antenna patterns show a substantial increase in photocurrent, reportedly enhancing it by 800% compared to other graphene‐based photodetectors. Moreover, the geometry of the plasmonic antenna plays a crucial role in determining the intensity of the photocurrent, due to the different scattering and absorption‐induced responsivity changes. Specifically, the heptamer configuration, for instance, offers a larger absorption cross‐section than the dimer, leading to enhanced field intensification, multiple hot spots, and a higher yield of hot electrons. Moreover, the nonamer configuration exhibits a pronounced Fano minimum, nearly 100%, markedly improving near‐field strengths and generating DE carriers in graphene. Therefore, the performance of 2D optoelectronic devices surpasses traditional materials, demonstrating application potential in next‐generation photodetectors, optical communication, and image sensors. Zhang et al. demonstrated a high‐performance artificial visual perception and recognition system using a plasmon‐enhanced 2D material neural network. This system integrates sensing, preprocessing and recognition functions, leading to significant improvements in the efficiency and accuracy of machine vision applications.^[^ ^148^ ^]^ When integrated with metallic nanostructures, plasmonic metasurfaces, waveguide‐coupled plasmons and nonlinear plasmonics, these 2D materials offer unique properties, leading to high‐performance, tunable, and compact plasmonic devices. Consequently, 2D materials demonstrate significant potential in optoelectronic modulators, microcavities, photovoltaics, and barrier photodetectors. However, challenges persist in achieving high‐quality, defect‐free 2D materials and minimizing defects at interfaces between 2D materials and traditional 3D materials. In summary, ongoing research and technological advancements are expected to address these challenges, paving the way for broader applications in future optoelectronic devices.

## 5 Integrations

**Figure 8:**
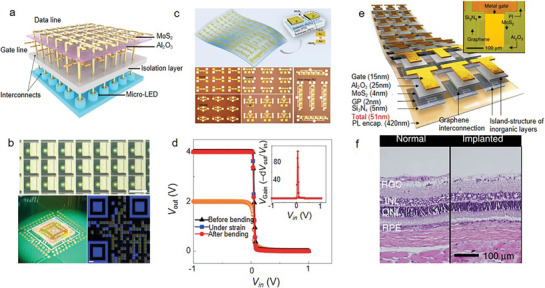
2D AM On‐chip Integrations applications. a) Schematic illustration of monolithic integration of MoS_2_ TFT s with micro‐LEDs. b) Optical micrographs of the active‐matrix micro‐LED display. The bottom picture represents a wire‐bonded chip viewed using an optical microscope, as well as microscopic pictures of the QR code shown on a 1270‐PPI blue micro‐LED display (Reproduced with permission.^[^ ^63^ ^]^ Copyright 2021, Springer Nature). c) Illustration of flexible transistor arrays with integrated circuits. Photographs of various MoS_2_ integrated devices on flexible substrates. d) Output voltage of an inverter as a function of input voltage when under different bending states. Inset: voltage gain of the inverter under an input of 4 V (Reproduced with permission.^[^ ^151^ ^]^ Copyright 2020, Springer Nature). e) A high‐density curved image sensor array based on MoS_2_‐graphene heterostructures is depicted schematically. f) The H&E stain histology of the normal retina and the retina implanted with the soft optoelectronic device (Reproduced with permission.^[^ ^62^ ^]^ Copyright 2022, Springer Nature).

2D AMs are a promising platform for engineering and modulating phonons and electrons at the nanoscale, which is highly important for integrated technologies. Kang et al. first fabricated high‐mobility 4‐inch wafer‐scale films of monolayer MoS_2_ and tungsten disulfide by using a new metal‐organic chemical vapor deposition technique. This technique enables the realization of a display made of large‐area TMDCs‐based FETs.^[^ ^149^ ^]^ The integration of 2D materials in micro‐LEDs has opened new avenues for enhancing their performance. Recently, Meng et al. demonstrated a 3D monolithic micro‐LED display comprising of large‐area MoS_2_ thin‐film transistors (TFTs) and GaN‐based micro‐LEDs (**Figure** **8**a).^[^ ^63^ ^]^ This innovative display technology enables the realization of micro‐level pixels that offer unparalleled brightness, with a 32 × 32 active‐matrix display achieving a remarkable density of 1270 pixels per inch. The display allows for addressing single pixels and high‐resolution Quick Response code images, evidenced by the microscopic images, as shown in Figure 8b. The median mobility of MoS_2_ transistors is 54 cm^2^ V^−1^ s^−1^ and a drive current of 210 µA µm^−1^, enabling them to drive micro‐LEDs to a luminance of 7.1 × 10⁷ cd m^−^^2^ at low voltages. Also, the higher mobility of 2D materials is beneficial to the low power consumption of the backplane. In addition, they reduce the gate voltage (*V_gs_*) and subthreshold slope (SS) by employing thinner or higher dielectric constant (*κ*) gate dielectrics. High drive current is achieved through depletion mode transistors. MoS_2_ transistors tune the brightness due to their high on/off ratio (10^9^). The bandwidth and pulse width modulation (PWM) optimize the brightness of micro‐LEDs, which meets the requirements for high‐dynamic‐range displays. A comprehensive analysis of the device performance indicates that MoS_2_ TFTs are exceptionally well‐suited for micro‐LED display applications, setting new limits for resolution and brightness. Furthermore, the integration of MoS_2_ in the back end of the line (BEOL) process, executed through a low‐temperature ultra‐clean method, ensures the preservation of MoS_2_ from external contamination. This approach minimizes the density of interface scattering centers, unintentional doping, and trap states, markedly enhancing device performance and uniformity across parameters such as mobility, SS, and threshold voltage. The technology that integrates the heterogeneous BEOL with mainstream semiconductor technology will spark 2D material technology in the future. Hwangbo et al. presented a novel active matrix micro‐LED display based on a MoS_2_ film directly grown on a GaN epitaxial wafer at low temperatures.^[^ ^150^ ^]^ This innovative approach allows for obtaining full‐color displays by overlaying quantum dots on blue micro‐LED, moreover, this technique not only facilitates scalable production but also enables the achievement of ultrahigh‐resolution displays. The key to this advancement lies in the seamless integration of MoS_2_ TFTs within the backplane of the micro‐LED display, eliminating the need for additional complex fabrication steps. Compared to TFTs backplane technologies, 2D materials display several advantages, such as crystalline materials (high mobility and atomically thin channel), wafer scale synthesis, BEOL integration with mainstream semiconductors, excellent flexibility, and optical transparency. The integration of 2D materials in micro‐LEDs offers unique properties, including high conversion efficiency and color purity and high‐resolution displays. This strategy not only develops a hybrid integration technique but also enhances the performance and flexibility of Micro‐LEDs.

Additionally, 2D materials such as graphene, transition metal dichalcogenides and black phosphorus have garnered significant attention for their potential applications in flexible circuits. Their unique electronic, optical and mechanical properties make them ideal candidates for next‐generation flexible electronic devices. Li et al. reported the transparent and flexible logic circuits based on wafer‐size MoS_2_ monolayers, including inverters, AND, NAND, NOR gates, ring oscillators and static random access memories (Figure 8c).^[^ ^151^ ^]^ These logic circuits show comparable performance with commercial ones, attributed to high mobilities and current densities, large on/off ratios of individual transistors. Herein, the low contact resistance plays a vital role in the improved device performance. The Au/Ti/Au contact maintains nice ohmic contact and adhesion between Au electrode and MoS_2_. At the same time, these devices not only demonstrate smaller subthreshold swing and higher on/off ratios than compared to Ti/Au‐contact devices, but also greatly reduce the Schottky barrier height (SBH). This leads to an ultra‐low contact resistance. Figure 8d shows the typical electrical characteristics of the inverter, with a high voltage gain under different voltages. This high gain is several times higher than in previous reports.^[^ ^152^, ^153^, ^154^ ^]^ Simultaneously, the voltage fluctuations of the flexible inverter are also tolerant to strain. By interfacing with reconfigurable circuits, the integrated detectors enable feedback and adaptive control, which are beneficial to deterministic quantum teleportation, neural networks training and circuit stability. Nevertheless, on‐chip integration faces challenges since the thermally reconfigurable photonic‐induced heat with heat‐sensitive single photon devices. To investigate compatibility, single‐photon detection, high‐extinction routing and quantum light, high‐dynamic‐range and stable input power have been investigated. Moreover, Choi et al. demonstrated a high‐density and semi‐spherical curved image sensor (CurvIS) array, which utilized 2D MoS_2_‐graphene heterostructures and strain‐releasing devices (Figure 8e).^[^ ^62^ ^]^ The CurvIS array ingeniously manages bending‐induced strains in rigid Si‐based detector arrays through a specially designed interconnection system, enabling wide field‐of‐view and aberration‐free imaging capabilities. Attributed to the ultrathin size and flexibility of 2D materials, the induced strain of the CurvIS array is far smaller than the fracture strain of the composite. To demonstrate the innovative technology, their authors propose a soft implantable optoelectronic device, inspired by the human eye that combines the CurvIS array with ultrathin neural‐interfacing electrodes, which design imitates the structural characteristics of the human eye. Such a design minimizes mechanical side effects, making it an ideal candidate for soft implantable applications. Histological comparisons depicted in Figure 8f between the implanted device and the normal retina show similar fibroblast growth and glial fibrillary acidic protein, indicating long‐term mechanical and biocompatibility. The integration of 2D materials in flexible circuits represents a notable advancement in flexible electronics, including stretchable, wearable electronics, flexible energy storage devices, sensors and optoelectronics. Furthermore, these combinations possess unique properties such as high on/off ratios, mobility, flexibility and device density. These 2D AMs pave a new pathway to promote their applications in computing, communication, sensing, and information storage.

**Figure 9:**
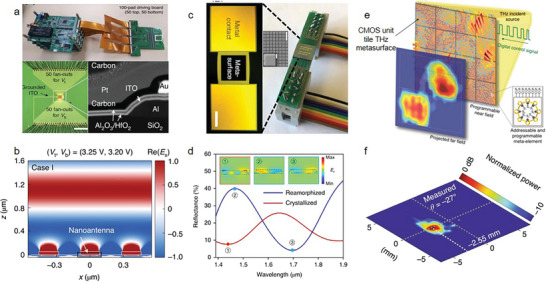
2D AM Integration applications. a) An active metasurface array that can drive 100 channels for 50 *V_t_* and 50 *V_b_* terminals separately is mounted on an electronics board. The 250 × 250 µm^2^ active array that located in the center of the fan‐outs is shown in the magnified view in the final figure. b) Simulated electric field (Re(*E_x_*)) distributions of the reflected light for (*V_t_*, *V_b_*) (Reproduced with permission.^[^ ^155^ ^]^ Copyright 2021, Springer Nature). c) An active metasurface device as seen through an optical microscope; scale bar, 100 µm. A wire‐bonded packaged metasurface array device on a custom‐designed PCB. Scanning electron microscopy (SEM) pictures of the metasurface are shown in the inset figure. d) Simulated reflectance spectra of the metasurface; insets plot the electric field in the z direction in a meta‐atom corresponding to the three states (1‐3) labelled on the spectra (Reproduced with permission.^[^ ^156^ ^]^ Copyright 2021, Springer Nature). e) Perspective images of the 2 × 2 tiled metasurface chips with quartz protection lid that were manufactured. A close‐up of the chip array and the packaging interface for voltage supply and digital control is shown at the bottom. f) Simulated and beamforming at −27° (Reproduced with permission.^[^ ^157^ ^]^ Copyright 2020, Springer Nature).

Metasurfaces are ultrathin, planar structures composed of subwavelength‐sized elements, that can manipulate electromagnetic waves with high precision. The integration of 2D materials into metasurfaces has opened new possibilities for controlling light at the nanoscale. Park et al. reported a reflective metasurface array with all‐solid‐state and electrically tunable properties. The array creates a particular phase or a successive sweep between 0 and 360° at ≈5.4 MHz while separately manipulating amplitudes.^[^ ^155^ ^]^ **Figure** **9**a shows that the active metasurface array installed on the electronic board. 100 channels are independently driven for 50 *V_t_* and 50 *V_b_* terminals. The active array at the center of the fanouts provides separate connections for 50 *V_t_* and 50 *V_b_* terminals. Figure 9b simulates the electric field (Re(*E_x_*)) distributions of reflected light under voltage combination (*V_t_*, *V_b_*), generating on‐demand reflection coefficients. By the independent superpositions of two input voltages, the amplitude and phase over 360° are independently tuned. In addition, a simulated street scene was presented by 3D depth scan, which includes a human picture and model car with a detection range >4.7 (not shown in Figure 9).

In addition, metasurfaces become a rising star in reconfigurable optics, due to increased diversity functionality, high compactness, and large manufacturability. Zhang et al. have made a notable contribution to this area with their development of an electrically reconfigurable non‐volatile metasurface platform depending on optical phase‐change materials, specifically highlighting the Ge_2_Sb_2_Se_4_Te (GSST) in their design (Figure 9c).^[^ ^156^ ^]^ The selection of meta‐atom dimensions is a critical aspect of their design, each meticulously designed to support both unique hybrid plasmonic‐photonic modes across the amorphous and crystalline states within a targeted wavelength range. The transition between these two states yields a substantial optical contrast at a wavelength of 1.49 µm as demonstrated in Figure 9d. In its crystalline state, the device efficiently couples to mode 1, while the coupling to mode 3 is significantly reduced in the amorphous state because of optical phase mismatch, which was confirmed theoretically. This breakthrough surpasses traditional phase‐change materials‐based active metasurfaces by achieving unprecedented non‐volatile index tuning capabilities, broadband operation, low optical losses, and extensive reversible switching volumes. Benefiting from these virtues, a record‐setting half‐octave spectral regime and an optical contrast greater than 400% was reported, setting new benchmarks for the performance of reconfigurable metasurfaces.

Venkatesh et al. made significant strides in the field of programmable metasurfaces by developing a large‐scale metasurface based on an innovative arrangement of CMOS chips, as illustrated in Figure 9e.^[^ ^157^ ^]^ An aperture comprised of a 2 × 2 tiled array CMOS chip and 576 metaelements was devised, where each metaelement could address and be programmed by 8 bits at GHz speed. The primary innovation here lies in the ability of this metasurface to facilitate phase transformation across its surface, enabling THz beamforming, as showcased in Figure 9f. The metasurface can dynamically reconfigure itself to direct THz beams in both azimuth and elevation directions, a feature that marks a significant advancement in the development of THz surface modulators. THz transmitters without frequency mixers were realized, meaning the results guide research into THz sensing and imaging, as well as multi‐gigabit‐per‐second THz wireless communication.

**Figure 10:**
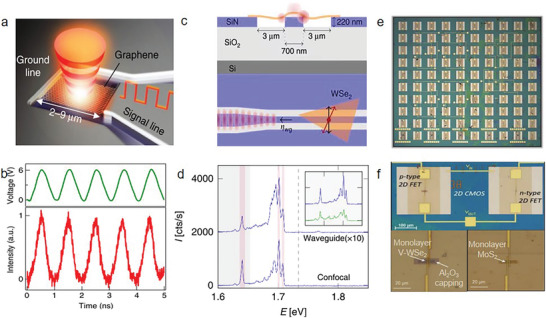
2D AM On‐chip Integrations applications. a) A schematic illustration of a graphene blackbody emitter. The square graphene sheet is connected to the source and drain to obtain modulated blackbody emission. b) The graphene blackbody emitter is measured by time‐resolved emission under continuous wave input voltage. The green curve represents the continuous input of the device at 1 Gbps (0–6 V height). The red curve represents its high‐speed emission modulation measurement (Reproduced with permission.^[^ ^158^ ^]^ Copyright 2018, Springer Nature). c) Integrated WSe_2_ quantum emitters. The upper part of the figure is the cross section of the sample, and the lower part is the top view of the device. A WSe_2_ flake is integrated on a single‐mode SiN waveguide (220 nm thick and 700 nm wide), which is separated from the bulk SiN by two air trenches (3 µm wide). To make the waveguide and the lensed fiber more easily coupled, it is designed to be tapered. The orientation of the dipole moment of the WSe_2_ emitters (red arrow) is random with respect to the quasi‐TE polarization (aligned approximately in the x‐direction) of the basic waveguide mode (black arrow). d) Confocal and waveguide‐coupled spectrum, excited with *λ* = 702 nm. Common peaks are highlighted by purple shaded areas. Filter the pump with a 715 nm (1.73 eV) long pass filter (shown by the dotted line). The inset figure shows confocal spectra obtained by either excitation with *λ* = 702 nm (blue curve) or excitation with *λ* = 532 nm (green curve) (Reproduced with permission.^[^ ^159^ ^]^ Copyright 2019, Springer Nature). e) The fully manufactured 2D CMOS chip. f) A 2D CMOS inverter and optical image of separate p‐type V‐WSe_2_ and n‐type MoS_2_ FET. Both the channel length and width of p‐type V‐WSe_2_ FET and n‐type MoS_2_ FET are designed as 1 and 5 µm, respectively. The necessary circuit connections between p‐type FET and n‐type FET are realized by using electron beam lithography to define 7 µm wide lines, and using electron beam deposition to evaporate 60 nm Ni/30 nm Au. (Reproduced with permission.^[^ ^165^ ^]^ Copyright 2022, Wiley‐VCH).

Miyoshi et al. reported ultrahigh‐speed (100‐ps response time), highly integrated graphene based blackbody emitters on‐Si‐chip, which locate in the near‐infrared region including wavelengths used for telecommunication.^[^ ^158^ ^]^ Both monolayer and few‐layer graphene can achieve impressive response times of approximately 100 ps, which translates to modulation speeds of ≈10 GHz. The schematic of the graphene blackbody device is depicted in **Figure** **10**a, showing the graphene linked to source and drain electrodes under a DC bias, enabling tuned blackbody emissions via the application of input signals. A high‐speed emission modulation from trilayer graphene under a continuous input of 1 Gbps by using time‐resolved emission measurements was obtained, as indicated in Figure 10b. The graphene emitterʼs response time is 100‐ps which not only surpasses the performance of conventional light‐emitting diodes but also rivals that of laser diodes operating in the GHz range. The mechanism behind this high‐speed modulation capability is due to fast thermal relaxation within the graphene, which is governed by traditional thermal transport mechanisms, including in‐plane and out‐of‐plane heat conduction, as well as distant quantum thermal transport through the surface polar phonons of the substrate materials. It provides a way to modulate the device's speed and the emission intensity through graphene's thickness and contact. Graphene will be important for integrated technologies with silicon chips, generating a smaller footprint for electronics and optoelectronics.

Photonic integrated circuits (PICs) not only scale down optical quantum circuits but also bring together multiple optoelectronic functionalities on a single chip. Recently, Peyskens et al. reported a single photon emitter with large single photon extraction, which integrates monolayer WSe_2_ onto a SiN chip (Figure 10c). Scaling down optical quantum circuits and consolidating multiple optoelectronic functionalities within a single platform is a significant step toward the realization of compact and efficient quantum photonic circuits. Both ends of the waveguide are tapered so that it is easy to couple with the lensed fiber. The PL of WSe_2_ is coupled with the light in free space and the waveguide's guided mode. Furthermore, the coupling with the cavity improves the guided modes’ overall coupling rate, optimizing the count rate of the integrated SPEs. The quantum properties of the emitters are meticulously analyzed, with confocal and waveguide coupling spectra demonstrating strong coupling between the emitters and the waveguide, evidenced by the appearance of distinct peaks shown in Figure 10d. A significant waveguide‐coupled peak occurs at ≈1.64 eV (756.5 nm), indicative of high electromagnetic overlaps near the waveguide mode center, leading to large integrated intensity. The several narrow peaks appear close to a spatial non‐uniformity in the WSe_2_ sheet, which may be due to cracks, wrinkles, or the transition between the monolayer and the bilayer. This spatial non‐uniformity usually creates strong strain gradient regions and they are highly related to the local bright spots including narrow linewidth emitters in monolayer TMDC. Therefore, the strain is most likely responsible for the various narrow peaks. Moreover, the 2D‐based quantum dots's PL intensity significantly increases as the excitation wavelength approaches the excitonic resonance, showing a substantial rise in intensity and a reduction in background noise under the same excitation power compared to λ = 532 nm.^[^ ^159^ ^]^ Integrated single photon emitters provide a promising route toward quantum scalable light sources and quantum communication through engineering strain, microstructures and defects.^[^ ^160^, ^161^, ^162^ ^]^

Zeng et al. reported pixel processing based on a single WSe_2_ transistor, showing multiple logic functions (AND and XNOR) that can be electrically switchable.^[^ ^163^ ^]^ These processing units are integrated into an image‐processing array utilizing the dual channel WSe_2_ transistors, characterized by their low power consumption. The 3 × 3 array is composed of two‐surface‐channel (TSC) WSe_2_ transistors and an Al_2_O_3_ dielectric acting as encapsulated layers. The array implements image crossing and image comparison. Due to the same image processing capacity, the image processing unit's consumption is less than 16% compared with traditional circuits. The ability to switch logic functions between AND and XNOR by merely adjusting drain voltage further underscores the versatility and efficiency of this single device in fulfilling pixel processing requirements. In addition, two image processing tasks (i.e., searching intersections or similarities between two images) are also completed. The design greatly reduces the circuit redundancy as well as improves the utilization of transistors. Meanwhile, Liu et al. also demonstrated the small‐footprint transistor architecture for photoswitching logic and in situ memory.^[^ ^164^ ^]^ A 2D single floating‐gate transistor, achieves a remarkable 50% reduction in logic gate area compared to conventional Si‐based architectures; moreover, the new device architecture overcomes the limitations of von Neumannʼs architecture.

The continuous miniaturization of CMOS technology has been a driving force behind the rapid advancement of electronics. However, the side effects of the decreasing dimensions and the physical separations for different functional units inhibit high performance, low cost, and high energy efficiency. Recently, Pendurthi et al. introduced a novel approach by integrating n‐type MoS_2_ and p‐type vanadium‐doped WSe_2_ FETs through a large area growth method, demonstrating a leap toward a non‐von Neumann CMOS platform that exhibits non‐volatile and analog memory storage capabilities (Figure 10e).^[^ ^165^ ^]^ Charges are efficiently trapped at the dielectric and 2D material interface, a mechanism that underpins its memory storage functionality. For p‐type FETs, the conductance increases with a larger positive voltage pulse (*V_p_*), while the opposite effect is seen in n‐type FETs, where conductance decreases. The p‐type FETs can transition from a low to a high resistance state through the application of a high negative voltage pulse (*V*_N_), which shifts the threshold voltage toward the negative side. Conversely, n‐type FETs exhibit the opposite behavior. This nuanced control over the conductance states of the FETs forms the foundation of the non‐von Neumann CMOS platform, which allows for the accurate placement positioning of p‐type and n‐type FETs using separated gate control on a SiO_2_/p^++^‐Si substrate and facilitates the implementation of 2D CMOS inverters (Figure 10f). The mobility and Schottky barrier of Ni/MoS_2_ interface are different from those of Ni/V‐WSe_2_ interface, which result in a high on‐current of n‐type MoS_2_ FETs compared with V‐WSe_2_ FETs. Astonishingly, the transmission characteristics exhibit a non‐monotonic behavior with exponential tails, reminiscent of a Gaussian distribution, enabling the realization of various digital and neuromorphic computing primitives. In addition, Zhu et al. presented high‐integration‐density 2D‐CMOS hybrid microchips for memristive applications by using multilayer hBN integrated onto the back‐end‐of‐line interconnections of silicon microchips (180 nm node).^[^ ^166^ ^]^ These hybrid 2D CMOS microchips achieve excellent electrical and endurance performance properties (5 million cycles despite their very small size 0.053 µm^2^). Also, in‐memory computing is realized and applied to spiking neural networks. Despite many challenges for practical microelectronic products, 2D CMOS shows tremendous potential in computing, memory, communication, artificial intelligence, and machine learning.

## 6 Conclusion and Outlook

This review summarizes the latest advancements in 2D AMs, including heterostructures, superlattices, metasurfaces, microcavities, etc. It discusses electronic, photonic, and optoelectronic devices in detail, highlighting the significant progress in efficient electrical and photonic property modulations, which benefit from enhanced light‐matter interactions. These advancements stimulate the design of high‐performance and multi‐functional 2D AM photonic and optoelectronic devices.

**Figure 11:**
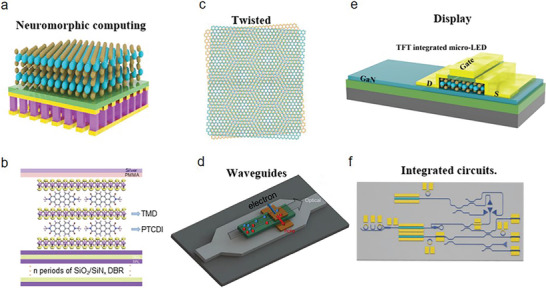
2D AM potential application and future developments. a) Schematic of 2D material heterostructures for neuromorphic computing. b) Schematic of organic‐inorganic superlattice microcavities. c) Schematic of similar‐structure 2D material layers with small angles. d) The schematic of all‐optical nonlinear units (AONUs). e) Schematic of integration of 2D materials/GaN in display applications. f) Integrated circuits encompassing superlattices, metasurfaces, microcavities.

For challenges and opportunities, the increased complexity and difficulties in both design and manufacture have become considerable roadblocks to achieving desired properties and functionalities. 2D AM electronic devices typically exhibit high mobility, high on/off ratio and low threshold voltage performance, making them suitable for applications in field‐effect transistors and memory devices. To further enhance device performance, improvements are mainly focused on electrode contacts, gate dielectrics, tunneling FET structures and spin FET structures. For example, 1) reducing contact resistance by using mechanically transferred electrodes and exploiting new contact metals, such as Sb(0001) and Bi(0001). 2) Utilizing low‐defect gate dielectrics, such as hBN or fluoride ion gate electrolytes, etc. Moreover, it is imperative to develop a universal and efficient method for fabricating various 2D AMs. For superlattices, the quality of constituent layers and interfaces heavily influence their properties, which are determined by complicated microfabrication processes (e.g., lithography, transfer process, electron beam evaporation). While stacking the similar‐structure 2D material layers with small, twisted angles, moiré patterns may form, showing great tunability of electrons.^[^ ^167^ ^]^ 3) In neuromorphic computing and memory devices mainly use the stacking of different 2D materials to form new heterostructures, providing scientific evidence for the application of parallel computing technology in the field of neuromorphic computing (**Figure** **11**a).

It is critical to fabricate the emission sources with an ultra‐narrow band spectral response by integrating 2D materials or heterostructures into optical cavities. Particularly, the microcavity of organic‐inorganic superlattices remains a virgin land, despite high absorption and strong optical emissions of organic counterparts (Figure 11b). This is because the synthesis and transfer method are not yet mature. For theoretical simulations, the relationship between microstructures and optoelectronic properties is still a mystery. While moiré patterns may form with the small twisted angles (similar‐structure 2D material layers), showing great tunability of photons and phonons. This provides a new avenue to manipulate and encode quantum systems, which is promising in the storage, process and transmission of quantum information (Figure 11c).^[^ ^168^ ^]^ In addition, twisted angle‐dependent moiré excitons offer additional tunability and distinct dipolar characteristics, which are beneficial for observing dipolar interaction‐induced classical quantum phases and novel phenomena.^[^ ^99^ ^]^ Concerning all‐optical neural networks, a challenge lies in the integration of 2D materials with Si photonics, due to the weaker nonlinear effect of the latter (Figure 11d).^[^ ^169^ ^]^

In the case of photodetectors, a clear opportunity lies in the exploration of high‐responsivity, ultrathin and high‐absorption 2D AM photodetectors, such as infrared photodetectors or terahertz photodetectors, polarization photodetectors, and so on. Infrared photodetectors based on 2D materials exhibit distinctive optoelectronic properties, including high sensitivity, low noise, broadband response, high detectivity and low dark current. However, further research is required in the material synthesis, manufacturing process and device structure optimization for 2D infrared detectors to facilitate mass production and commercialization. Moreover, 2D THz photodetectors hold significant potential for integration with resonant antennas and metasurfaces. Meanwhile, there is substantial research interest in polarization‐sensitive detectors based on anisotropic 2D materials. However, ongoing efforts are required to enhance key parameters such as response wavelength, photoresponsivity, response time and anisotropy ratio. Reports on active metasurfaces are scarce at the current stage, which hinders the dynamic control of optoelectronic properties using external stimuli. Moreover, dispersionless flat lenses have emerged as a promising research direction. These lenses could correct chromatic aberration throughout a wide spectral range, reducing coma spherical aberration and other monochromatic aberrations, which overturn the current optical apparatus. Also, radiative‐cooling 2D AMs (e.g., metasurfaces) have attracted significant attention due to their distinct thermoregulatory properties. Furthermore, the new material research for 2D AMs that have lower losses, higher melting points, excellent modulation and CMOS compatibility will be attractive in the future.

Regarding integration, the primary research focuses include the monolithic integration of neuropeptide sensors, 2D flexible electronics and integration of 2D materials with silicon. 2D neuropeptide sensors exhibit high sensitivity, fast response, flexibility and stretchability, making them applicable to wearable and biomedical devices. However, ensuring the biocompatibility and safety of 2D materials for biomedical applications is essential to avoid potential adverse effects on biological tissues. Meanwhile, 2D flexible devices show great potential for applications in flexible displays and wearable devices, primarily due to the advantages of 2D materials being lightweight, thin, highly transparent and high mechanical strength. Further improvement is needed in the stability of 2D materials under continuous bending, stretching and environmental exposures. Additionally, the cost‐effective and scalable production of 2D materials is essential for their widespread adoption in flexible electronics. Integrating 2D materials with silicon can enhance performance and facilitate mass production by leveraging the unique electronic, optical, and mechanical properties of the former, in combination with the well‐established silicon manufacturing process. Nevertheless, the primary challenge lies in the monolithic 3D integration with 2D AMs (Figure 11e), attributed to the lack of large‐scale 2D AMs that show the consistency of structure and electrical/optoelectronic properties (Figure 11f). These immature manufacturing technologies heavily influence academic and industrial research.

Despite many challenges, 2D AMs exhibit a bright future in both the academic and commercial fields, benefiting from the virtues, such as efficient tunings, enhanced light‐matter interactions, high integrations, low power consumption and low dimensions. They also provide a powerful platform to achieve high‐performance and novel functional devices that are beyond natural materials and systems. This review calls for more researchers to join us to completely exploit the potential of 2D AMs in optoelectronics.

## Conflict of Interest

The authors declare no conflict of interest.
